# Supplementary material for: A Global Screen for Assembly State Changes of the Mitotic Proteome by SEC-SWATH-MS
Source: Cell Syst. 2020 Feb 26;10(2):133–155.e6. doi: 10.1016/j.cels.2020.01.001 (PMC7042714; doi:10.1016/j.cels.2020.01.001)

Q6ZSZ5 | ARHGI_HUMAN | ARHGEF18 KIAA0521
Monomer MW [kDa]: 130.78 Monomer expected elution fraction: 38

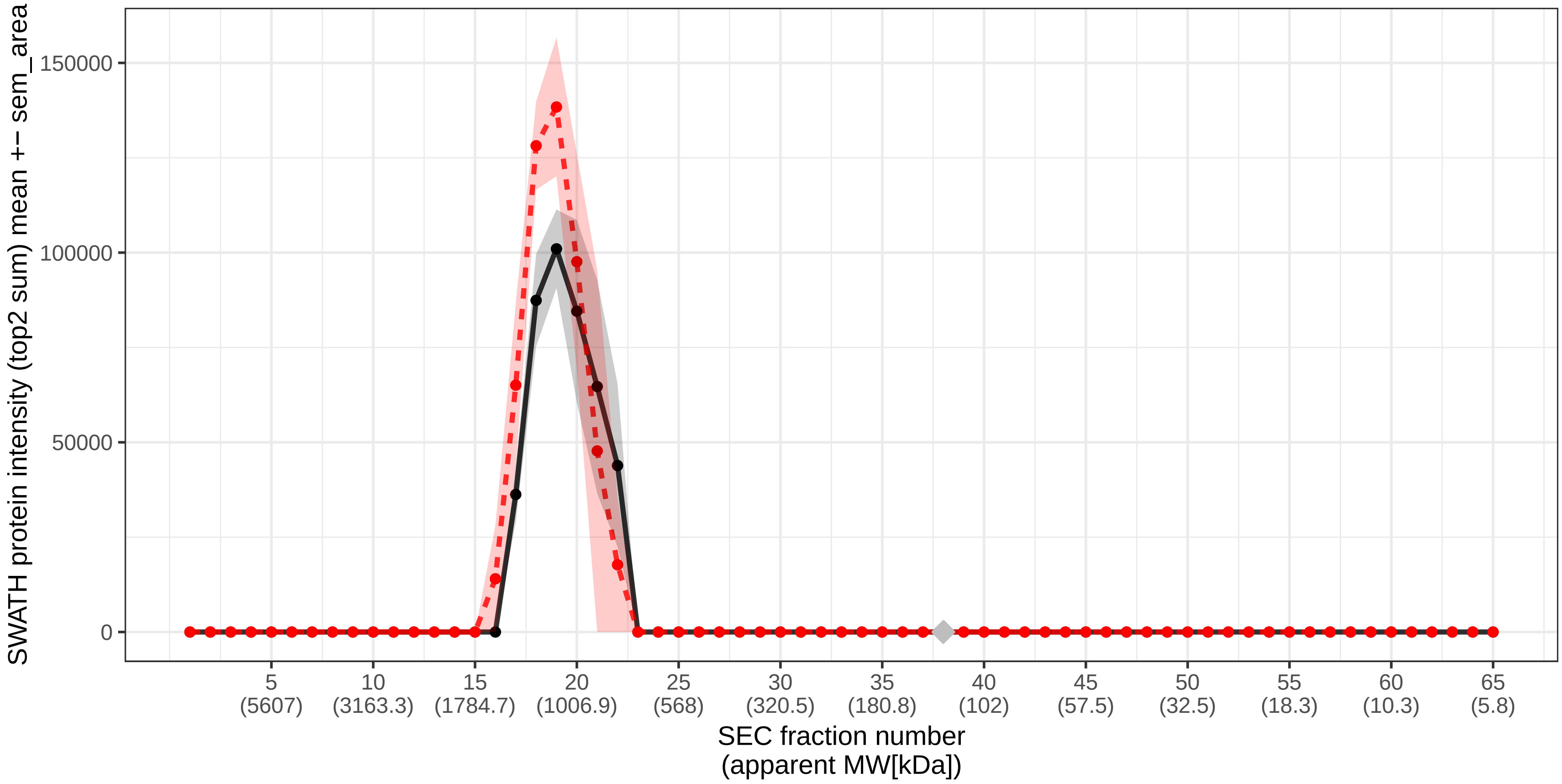

Supplement: Data S1. SEC-SWATH-MS Protein Chromatograms, Related to Figure 1 [file mmc6.zip › SECchrom_Q6ZSZ5_ARHGI_HUMAN_ARHGEF18_KIAA0521.pdf]
